# TcSR62, an RNA-binding protein, as a new potential target for anti-trypanocidal agents

**DOI:** 10.3389/fmicb.2025.1539778

**Published:** 2025-03-12

**Authors:** Analía G. Níttolo, Agustina M. Chidichimo, Ana L. Benacerraf, Timothy Cardozo, M. Clara Corso, Valeria Tekiel, Javier G. De Gaudenzi, Gabriela Vanesa Levy

**Affiliations:** ^1^Comisión de Investigaciones Científicas de la Provincia de Buenos Aires (CIC), La Plata, Argentina; ^2^Departamento de Ciencias de la Salud, Universidad Nacional de La Matanza, San Justo, Argentina; ^3^Instituto de Investigaciones Biotecnológicas, Universidad Nacional de San Martín (UNSAM) - Consejo Nacional de Investigaciones Científicas y Técnicas (CONICET), San Martin, Argentina; ^4^Escuela de Bio y Nanotecnologías (EByN), Universidad Nacional de San Martín, San Martin, Argentina; ^5^NYU Langone Health, New York University School of Medicine, New York, NY, United States

**Keywords:** Chagas disease, *Trypanosoma cruzi*, treatment, sorafenib tosylate, molecular modeling, resazurin, IC50, drug repurposing

## Abstract

Trypanosomatids are parasites of health importance that cause neglected diseases in humans and animals. Chagas’ disease, caused by *Trypanosoma cruzi*, affects 6–7 millions of people worldwide, mostly in Latin America, most of whom do not have access to diagnosis or treatment. Currently, there are no available vaccines, and the antiparasitic drugs used for treatment are often toxic and ineffective for the chronic stage of infection. Therefore, exploration of new therapeutic targets is necessary and highlights the importance of identifying new therapeutic options for the treatment of this disease. Trypanosomatid genes are organized and expressed in a species-specific fashion and many of their regulatory factors remain to be explored, so proteins involved in the regulation of gene expression are interesting candidates as drug targets. Previously, we demonstrated that the TbRRM1 protein from *T. brucei* is an essential nuclear factor involved in Pol-II transcriptional regulation. TcSR62 is a TbRRM1 orthologous protein in *T. cruzi*, but little is known about its function. In this study, we used molecular modeling of the RNA-binding domains of the TcSR62 protein and computational molecular docking to identify TcSR62-specific drug candidates. We identified sorafenib tosylate (ST) as a compound with trypanocidal activity. Sorafenib tosylate showed promising half-maximal inhibitory concentration (IC50) for all parasite stages *in vitro*. Furthermore, overexpression of TcSR62 protein led to ST-resistant parasites, suggesting that the trypanocidal effect might be due to the inhibition of TcSR62 function. These results demonstrate that ST could be repurposed as a novel drug to treat Chagas’ disease.

## 1 Introduction

The human pathogen *Trypanosoma cruzi* (Chagas, 1909) (Kinetoplastida; Trypanosomatidae) is the causative agent of Chagas’ disease (CD), an endemic illness in Latin America (World Health Organization, 2024). The life cycle of the parasite includes both a vertebrate host of the class Mammalia and a vector insect belonging to the Triatominae subfamily, with different developmental stages in each host. *Trypanosoma cruzi* infection in humans is established by the infective form of metacyclic trypomastigotes. It invades host cells and differentiates into a replicative amastigote form, which then transforms into bloodstream trypomastigotes. This stage can circulate in the blood, infecting other cells or be eventually ingested by blood-feeding insect during its meal. Inside the vector, ingested trypomastigotes differentiate into replicative epimastigotes. This form migrates along the digestive tract until it differentiates into metacyclic trypomastigotes, which are released onto the skin or mucous membranes along with triatomine feces/urine, thus closing the cycle (Ferreira et al., 2023).

Trypanosomes are unicellular eukaryotes that have particular features in terms of mechanisms leading to protein expression (Rodrigues et al., 2014). One of these is the mechanism of transcription and mRNA processing. In these organisms, mRNAs are initially produced as polycistronic units that subsequently undergo processing to generate mature mRNAs (Romagnoli et al., 2020; De Gaudenzi et al., 2011). Several RNA-binding proteins (RBPs) have been studied because of their role as key factors in gene expression regulation, including serine/arginine-rich (SR) proteins. They are involved in processes such as RNA transcription, processing, export, and the control of mRNA stability (Zhong et al., 2009; Jeong, 2017). The “canonical” SR proteins are highly conserved with a modular domain structure consisting of one or two amino-terminal RNA Recognition Motifs (RRMs), and a carboxyl-terminal R/S region rich in Arg-Ser dipeptides.

*Trypanosoma cruzi* SR62 (TcSR62, TcCLB.511621.50) and *Trypanosoma brucei* (Plimmer and Bradford, 1899) RRM1 (TbRRM1, Tb927.2.4710) are orthologous proteins from the SR-related family, featuring three RRMs, two Cys2-His-Cys-type zinc fingers, and one C-terminal R/S domain, with non-mammalian orthologs.

Our previous results demonstrated that TbRRM1 is essential in both procyclic and bloodstream trypomastigotes, suggesting its crucial role in parasite biology (Levy et al., 2015; Níttolo et al., 2018). Although there are several reports about the function of TbRRM1, known to regulate both transcriptional and post-transcriptional processes in *T. brucei* (Levy et al., 2015; Bañuelos et al., 2019; Naguleswaran et al., 2015) little information is available regarding the function of TcSR62. Proteomic studies suggest that it may associate with chromatin (Leandro de Jesus et al., 2017), localized in reservosomes (Ulrich et al., 2011), and the plasma membrane (Queiroz et al., 2013; Cordero et al., 2009), indicating potential roles beyond those currently known for TbRRM1. TcSR62 mRNA has been identified in *T. cruzi* extracellular vesicles implying its significance in host-pathogen interaction (Fernandez-Calero et al., 2015). These striking findings suggest that this protein may play a relevant role in *T. cruzi* infection; however, none of these results have been validated. TcSR62 shows major nuclear localization in epimastigotes of the CL-Brener strain, but its expression in trypomastigotes, amastigotes, or other *T. cruzi* strains remains unstudied (Názer et al., 2011). In epimastigotes, previous studies have shown that under transcriptional stress or severe heat shock, TcSR62 relocalizes to the nucleolus, along with other known RBPs suggesting a stress response function (Názer et al., 2011; Názer et al., 2012).

While vectorial transmission is the primary route of *T. cruzi* infection, blood transfusions, organ transplants, congenital transmission and even food contamination (Simões-Neto et al., 2024) are other ways of acquiring the disease. It is estimated that 6–7 million people worldwide, mostly in Latin America are infected with this protozoan parasite, and that 75 million inhabitants (around 18% of the region’s total population) are at risk of contracting the disease (World Health Organization, 2024; Echeverría et al., 2020). In addition, it was estimated that over 80% of individuals worldwide affected by CD lack proper access to diagnosis and treatment, leading to increased patient morbidity and mortality rates (Pinheiro et al., 2017). Chagas’ disease presents in two stages: an acute stage, which, in most cases, is asymptomatic, and a chronic phase, in which approximately one-third of the patients may develop cardiac, digestive and/or neurological dysfunctions (Malik et al., 2015; Medina-Rincón et al., 2021; Nunes et al., 2018; Useche et al., 2022). Benznidazole (BNZ) and nifurtimox are two antiparasitic drugs used to treat this disease. Nifurtimox is intermittently available and has been proposed as a second-line treatment in Argentina, Brazil, Chile and Uruguay (Pérez-Molina et al., 2020; Miranda-Arboleda et al., 2021). Already BNZ is effective in the acute phase, with several reported adverse effects in adults, such as dermatologic, gastrointestinal and neurological side effects, leading to treatment interruption in a large number of cases (Malik et al., 2015; Franco et al., 2021). Benznidazole has been reported to be fully effective against *T. cruzi* amastigotes at high doses *in vivo* (Bustamante et al., 2020) however its therapy has little benefits (Pérez-Molina and Molina, 2018). There are still limitations in its treatment because BNZ therapy did not significantly reduce cardiac clinical deterioration of chronically infected patients (Morillo et al., 2015) and because of its adverse effects (Crespillo-Andújar et al., 2022; Hasslocher-Moreno, 2024). Although trypanocidal therapy was effective in eliminating the congenital transmission of *T. cruzi* in infected women at reproductive age (De et al., 2024), new therapeutic approaches as monotherapy or in combination with BNZ are urgently needed (Ramos et al., 2024).

To evaluate TcSR62 protein as a potential repurposing target for anti-trypanocidal agents, we confirmed its expression pattern and cellular localization in different stages and strains. A computational molecular docking screen of an FDA-approved drug library identified several promising candidates that exhibited strong affinity for TcSR62. Following this, we aim to determine which of these drugs was the most effective compound against *in vitro* cultured parasites.

## 2 Materials and methods

### 2.1 Compounds

Several drugs from Table 1 were selected based on their docking score and availability, as well as others that were selected for presenting highly predictive interactions to the pockets of RRM2 and RRM3. Benserazide, roflumilast, sorafenib tosylate, celiprolol hydrochloride, fludarabine, cefoxitin sodium, lonidamine, pyrantel pamoate, lafutidine, midodrine hydrochloride and nepafenac were acquired from Sigma-Aldrich. Paclitaxel and daunorubicin were obtained from Varifarma Laboratory. Depending on their solubility, the compounds were resuspended in dimethyl sulfoxide (DMSO) or water at a stock concentration of 40 mM. Benznidazole, obtained from Elea Laboratory, was used as the gold standard.

**Table 1:** Compounds that bound to the modeled RRM1 domain of TcSR62, with their docking score calculated by ICM-VLS.

Compound	CAS	Calculated free energy
Pyrantel pamoate	22204-24-6	−23.96
Lyothyronine	6893-02-3	−23.02
Vidarabine	5536-17-4	−22.15
Nepafenac	78281-72-8	−21.37
Dicloxacillin sodium	13412-64-1	−20.47
Roflumilast	162401-32-3	−20.10
Amrinone	60719-84-8	−19.84
Midodrine HCl	3092-17-9	−19.65
Dipivefrin HCl	64019-93-8	−19.59
Flumethasone	2135-17-3	−19.25
Sorafenib tosylate	475207-59-1	−19.20
Trifluridine	70-00-8	−19.18
Benserazide HCl	14919-77-8	−18.54
Salifungin	3679-64-9	−18.30
Ruxolitinib phosphate	1092939-17-7	−17.96

### 2.2 Parasite cultures

Epimastigotes of the Dm28c *T. cruzi* I (TcI), Ac (TcI) (Risso et al., 2004), Sylvio (TcI), Y (TcII), CL-Brener (TcVI), and RA (TcVI) strains were cultured at 28°C in BHT medium containing brain heart infusion (Sigma-Aldrich), 0.3% tryptose, 0.002% bovine hemin and 10% heat-inactivated fetal bovine serum (FBS) (BHT 10%).

Cell-derived *T. cruzi* trypomastigotes of Dm28c, Ac, Sylvio, Y, CL-Brener, RA, and Tulahuen (TcVI) β-galactosidase (Tul β-gal) (Buckner et al., 1996) were cultured by passages in Vero cells at 37°C in a humidified atmosphere containing 5% CO_2_ in minimum essential medium (MEM; Gibco Life Technologies) supplemented with 10% FBS, 10 μg/mL streptomycin and 100 U/mL penicillin. Trypomastigotes were harvested from the supernatants of infected cells at 5,000 × g for 10 min after 96 h post-infection. For the amastigote stage, the cultures were maintained for 10 days in 4% FBS in MEM, then harvested by centrifugation at 100 × g for 10 min to remove the detached cells and finally for additional 10 min at 5,000 × g to collect the amastigote form.

### 2.3 Western blot assays

Epimastigotes (5 × 10^6^), trypomastigotes (1 × 10^7^) and amastigotes (1.5 × 10^7^) from different strains of *T. cruzi* were harvested by centrifugation at 1,620 × g, washed in phosphate-buffered saline (PBS), resuspended in cracking buffer (50 mM Tris pH 6.8, 0.1 M DTT, 2% SDS w/v, 0.1% bromophenol blue, and 10% glycerol), and boiled for 5 min. Total protein extracts were separated on a 12% acrylamide-bisacrylamide 29:1 gel using a Mini Protean vertical electrophoresis chamber (Bio-Rad, Hercules, CA, United States) in running buffer containing 25 mM Tris, 192 mM glycine, and 0.1% SDS w/v at a final pH of 8.3 at 15 V/cm. After running the gel, the proteins were transferred to a nitrocellulose membrane (Amersham Hybond^™^-ECL, GE Healthcare) at 0.2 A using a Miniprotean II transfer chamber (Bio-Rad) in transfer buffer (25 mM Tris, 190 mM glycine, and 20% methanol at a final pH of 8.3). The membranes were incubated overnight in a cold chamber at 4°C in 5% non-fat milk and then incubated with a serum that specifically recognizes the TcSR62 protein made in rabbit and anti-β tubulin made in mouse. Anti-rabbit or anti-mouse conjugated to IRDye 680LT and 800CW, respectively (LI-COR), were used as secondary antibodies. Protein detection was performed using the Oddisey DLx Imaging System (LI-COR Biosciences). Protein quantification was performed by ImageJ software and normalized to enolase or β-tubulin expression levels. For the TcSR62 overexpression assay, anti-FLAG monoclonal antibody (Sigma-Aldrich), anti-SR62 rabbit serum or anti-enolase rabbit serum were used as primary antibodies.

### 2.4 Indirect immunofluorescence assay

The subcellular localization of TcSR62 was evaluated by indirect immunofluorescence (IIF) in permeabilized epimastigotes, trypomastigotes and amastigotes from different *T. cruzi* strains. For this, 5 × 10^6^ epimastigote-stage parasites and 1 × 10^7^ trypomastigote were centrifuged at 1,620 × g, washed twice in PBS and placed on coverslips previously treated with 0.25 mg/mL poly-L-lysine (Sigma-Aldrich) for 20 min. For amastigotes, 1 × 10^4^ Vero cells were cultured on coverslips in MEM 10% FBS for 48 h and infected with different strains of *T. cruzi* trypomastigotes at a multiplicity of infection (MOI) = 100 (CL-Brener and Y), MOI = 20 (for RA, Sylvio and Ac parasites) or MOI = 10 (for Dm28c and Tul β-gal strains). The samples were fixed in 4% paraformaldehyde (PFA, Electron Microscopy Sciences) in PBS for 20 min at room temperature. After washing with PBS, the samples were treated with 25 mM NH_4_Cl (Sigma-Aldrich) in PBS for 20 min, followed by blocking and permeabilization with a solution containing 2% BSA (Fedesa), 2% normal goat serum (Sigma-Aldrich), and 0.5% saponin (Sigma-Aldrich). After incubation for 1 h at room temperature, samples were washed with PBS and incubated with anti-SR62 serum for 1 h at 37°C in a humidified chamber. After washing the samples in PBS, secondary anti-rabbit IgG conjugated to Alexa 594 antibody (Life Technologies) was added, and incubation proceeded for 1 h at 37°C in a humidified chamber. The samples were mounted in Fluorsave (Merck) containing 100 ng/μL of 4′,6-diamidino-2-phenylindole (DAPI, Invitrogen) for visualization of nuclei and kinetoplasts. As negative control, parasites incubated with only secondary antibodies were used. The samples were visualized using a Nikon 80i direct fluorescence microscope with a CCD camera and LED illumination.

For the TcSR62 overexpression assay, anti-FLAG monoclonal antibody (Sigma-Aldrich) and anti-TcSR62 rabbit serum were used as primary antibodies and anti-mouse Alexa 488 and anti-rabbit Alexa 594 were used as secondary antibody (Life Technologies).

### 2.5 TcSR62 molecular modeling and docking

A close human structural homolog of the TcSR62 domain (PDB ID 5en1) has been resolved at high resolution by X-ray diffraction (~2.58 angstroms) (Wu et al., 2018). The sequence identity between this human template and the *T. cruzi* TcSR62 RRM domains is ~23%, but more importantly the ZEGA probability (Abagyan and Batalov, 1997) of major structural deviation between the sequences of the two domains is 10e^−8^, which means that the homology could occur by random chance between two structurally unrelated proteins in one out of 10^8^ randomly chosen pairwise alignments. This represents sufficient structural similarity to construct a high-quality homology model of TcSR62 using previously published methods (Cardozo et al., 1995). Briefly, the ZEGA global sequence alignment between the PDB 5en1 template and each of the three RRM domains was generated and the inserted and deleted amino acids forming loops were adjusted to structurally peripheral positions. This 3D model of each RRM domain was then built onto the PDB 5en1 template according to the alignment and energy minimized to remove van der Waals clashes and optimize electrostatic energy. A drug-binding pocket (Kufareva et al., 2012) (solvent cavity significantly enclosed by the 3D structure) with volume from 115.5 angstroms was observed on the first RRM domain (RRM1). We therefore used computational molecular docking to virtually screen the library of U.S. FDA approved drugs against this pocket using ICM-VLS (Molsoft, LLC, La Jolla CA) and hits were identified as significant by previously published criteria (Schapira et al., 2003). Molecular modeling and docking were done on a computer with an Intel^®^ Core^™^ i7-3770 CPU with 24GB RAM and UBUNTU Linux 14.04.

### 2.6 Viability assay with resazurin

To determine parasite viability, 1 × 10^6^ trypomastigotes/mL or 1 × 10^7^ epimastigotes/mL were incubated for 24 h or 48 h, respectively, with 10 μM of each compound, except for paclitaxel, which was incubated at 7 μM. DMSO or water (0.5% v/v) and 20 μM BNZ were used as controls. After incubation, 100 μL of parasites were transferred in triplicate to black 96-well plates and incubated with 44 μM resazurin for 4 h at 37°C for trypomastigotes and 28°C for epimastigotes in the dark (Rolón et al., 2006). After incubation, the emitted fluorescence was measured using a Beckman Coulter DTX 800 plate reader at 535–595 nm excitation-emission, respectively. The culture medium containing resazurin was used as a control for baseline fluorescence, and this was subsequently subtracted from the fluorescence readings of the entire plate. The measurements were normalized to vehicle controls (DMSO or water).

### 2.7 IC50 determination

The inhibitory concentration 50 (IC50) of sorafenib tosylate (ST) was calculated *in vitro* in trypomastigotes of RA and Ac strains and in epimastigotes of Dm28c expressing pLew13 vector. Serial dilutions of ST were performed, and viability at each point was determined using the resazurin assay, as previously described (Rolón et al., 2006). For Ac trypomastigotes, ST concentrations ranging from 0.001–10 μM were used. For trypomastigotes of RA strain, concentrations varied from 0.1 μM to 100 μM. For metacyclic parasites, ST was assayed from 1.25 to 50 μM, and for epimastigotes, ST concentrations ranged from 0.5 to 100 μM. For TcSR62 overexpressing parasites, ST concentrations from 0.001 μM to 50 μM were used in trypomastigotes and from 0.5 μM to 100 μM in epimastigotes. IC50 was calculated using GraphPad Prism 9 software, fitting a dose–response curve using the variable slope (four-parameter) model. DMSO 0.5% and the culture medium were used as negative controls.

The IC50 of ST at the Y metacyclic stage was determined in aged epimastigote cultures incubated with different concentrations of ST. Metacyclogenesis was calculated by counting metacyclic trypomastigotes in a Neubauer chamber.

### 2.8 Viability and infectivity after sorafenib tosylate treatment

Vero cells (1 × 10^4^) were seeded in 24-well plates with coverslips. After 24 h, infection was performed with trypomastigotes of the CL-Brener strain (MOI = 100). At 24 h post-infection, the wells were washed with PBS, and different concentrations of ST or 0.5% DMSO (vehicle) were added. The plates were then incubated for additional 72 h at 37°C in 5% CO_2_. Then, coverslips were washed with PBS, fixed with 4% PFA, and stained with DAPI for visualization under a fluorescence microscope. Viability/infectivity and the number of amastigotes per cell were determined by counting at least 900 cells per treatment in three independent biological replicates. The endocytic index was determined as the percentage of infected cells multiplied by the number of parasites per cell, as previously described (Bourguignon et al., 2006).

Alternatively, to determine the IC50 of ST on amastigotes, infections were performed with Tulahuen trypomastigotes expressing the β-galactosidase enzyme (Tul β-gal) (Vega et al., 2005). Vero cells (5 × 10^4^ per well) were seeded in a 96-well plate and after 48 h, infection was performed with Tul β-gal trypomastigotes (MOI = 10). After 24 h, the wells were washed with PBS, and different concentrations of ST were added in triplicate in 4% FBS RPMI medium without phenol red (Gibco). DMSO 0.5% and BNZ 20 μM were used as controls. After 72 h, 100 μL of PBS solution containing 0.5% NP40 and 50 μM chlorophenol red β-D-galactopyranoside (CPRG from Roche) were added and incubated for 4 h at 37°C. The absorbance was measured at 590 nm using a FilterMax F5 Multi-Mode microplate reader.

### 2.9 Cytotoxicity assays

Vero and H9C2 cells were grown in 96-well plates at 37°C in a 5% CO_2_ atmosphere in MEM or DMEM (Gibco) supplemented with 10% FBS (Natocor), respectively. Cells were cultured in the presence of 10 μg/mL streptomycin and 100 U/mL penicillin. To determine the cytotoxic effect of ST, cells were incubated with different concentrations of the compound and DMSO 0.5% was used as control. After 72 h of incubation, 44 μM resazurin was added to the cells. After 7 h of incubation at 37°C, 100 μL from each well were transferred in quintuplicate to black 96-well plates. Emitted fluorescence was measured using a Beckman Coulter DTX 800 Multi-mode Detector. The culture medium containing resazurin was used as a control for baseline fluorescence. The measurements were normalized to vehicle control (DMSO 0.5%). The Selectivity Index (SI) was calculated as the CC50/IC50 ratio (Nesic de Freitas et al., 2023).

### 2.10 3xFLAG-TcSR62 overexpressing parasites

Primers were designed to amplify the coding region of TcSR62 (ID TcCLB.511621.50b), which was tagged at the N-terminal end with 3xFLAG. The 3xFLAG-TcSR62 construct was cloned into the pGEM-T® easy vector (Promega) and, after sequencing, cloned into the tetracycline-inducible vector pTcINDEX using *Bam*HI sites (Taylor and Kelly, 2006). The construct was linearized using the *SpeI* restriction enzyme. Dm28c epimastigote parasites previously transfected with the pLew13 vector, which expresses T7 RNA polymerase and tetracycline repressor, were used for transfection. Briefly, 1×10^8^ epimastigote parasites grown in exponential phase were harvested at 1,620 × g, washed, and resuspended in 400 μL of Tb-BSF buffer, with 10 μg of DNA or water as control. The samples were transferred to 2 mm gap electroporation cuvettes for the Amaxa Nucleofector 2 B system using program U-033 (Pacheco-Lugo et al., 2017). Parasites were recovered in fresh culture medium, selected using 300 μg/mL hygromycin and cloned by limited dilution. Trypomastigotes containing the 3xFLAG-TcSR62 pTcINDEX were obtained as described above. For the epimastigote stage, induction was performed with 1 μg/mL tetracycline (TET) at a density of 3 × 10^7^ parasites for 24 h. For trypomastigotes overexpressing 3xFLAG-TcSR62, induction was performed for 3 h at a density of 1 × 10^6^ parasites.

## 3 Results

### 3.1 TcSR62 protein was expressed at all stages and in all *Trypanosoma cruzi* strains tested

**Figure 1:**
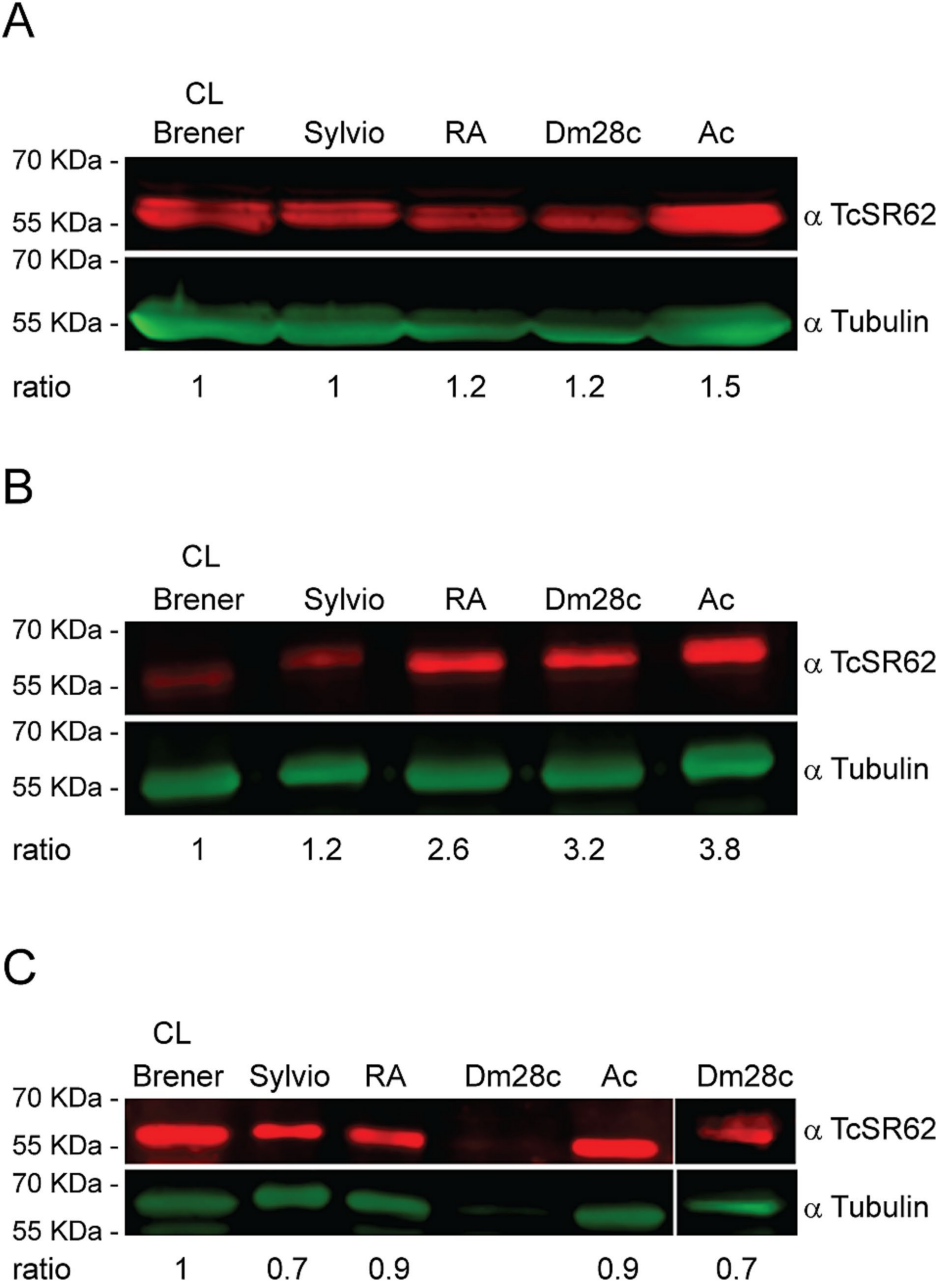
TcSR62 protein expression. Western blot assay of the trypomastigote **(A)**, epimastigote **(B)**, and amastigote **(C)** stages of different *T. cruzi* strains. An additional western blot assay was performed for Dm28c strain with 50 × 10^6^ amastigotes (**C**, right panel). Anti-tubulin antibody was used as a loading control. Protein detection was performed using the Odyssey DLx Imaging System (LI-COR Biosciences). Numbers indicate the normalized TcSR62 levels.

The expression levels of *T. cruzi* TcSR62 protein were investigated by western blotting in trypomastigotes, epimastigotes, and amastigote stage parasites from five distinct strains belonging to discrete typing units (DTU) TcI and TcVI. TcSR62 was widely expressed in trypomastigotes (Figure 1A), epimastigotes (Figure 1B) and amastigotes (Figure 1C) as a protein between 55–70 KDa. Parasites from Ac strain showed the highest levels of TcSR62 expression in both trypomastigotes and epimastigotes, whereas CL-Brener parasites showed the lowest levels of TcSR62 expression (Figures 1A,B). On the other hand, the lowest levels of TcSR62 expression were observed in amastigotes from Sylvio and Dm28c strains (Figure 1C). These findings indicate that TcSR62 was expressed in all strains and stages, although at different levels, suggesting that it may play an important role in the *T. cruzi* biology.

### 3.2 TcSR62 was mainly localized in the nucleus

**Figure 2:**
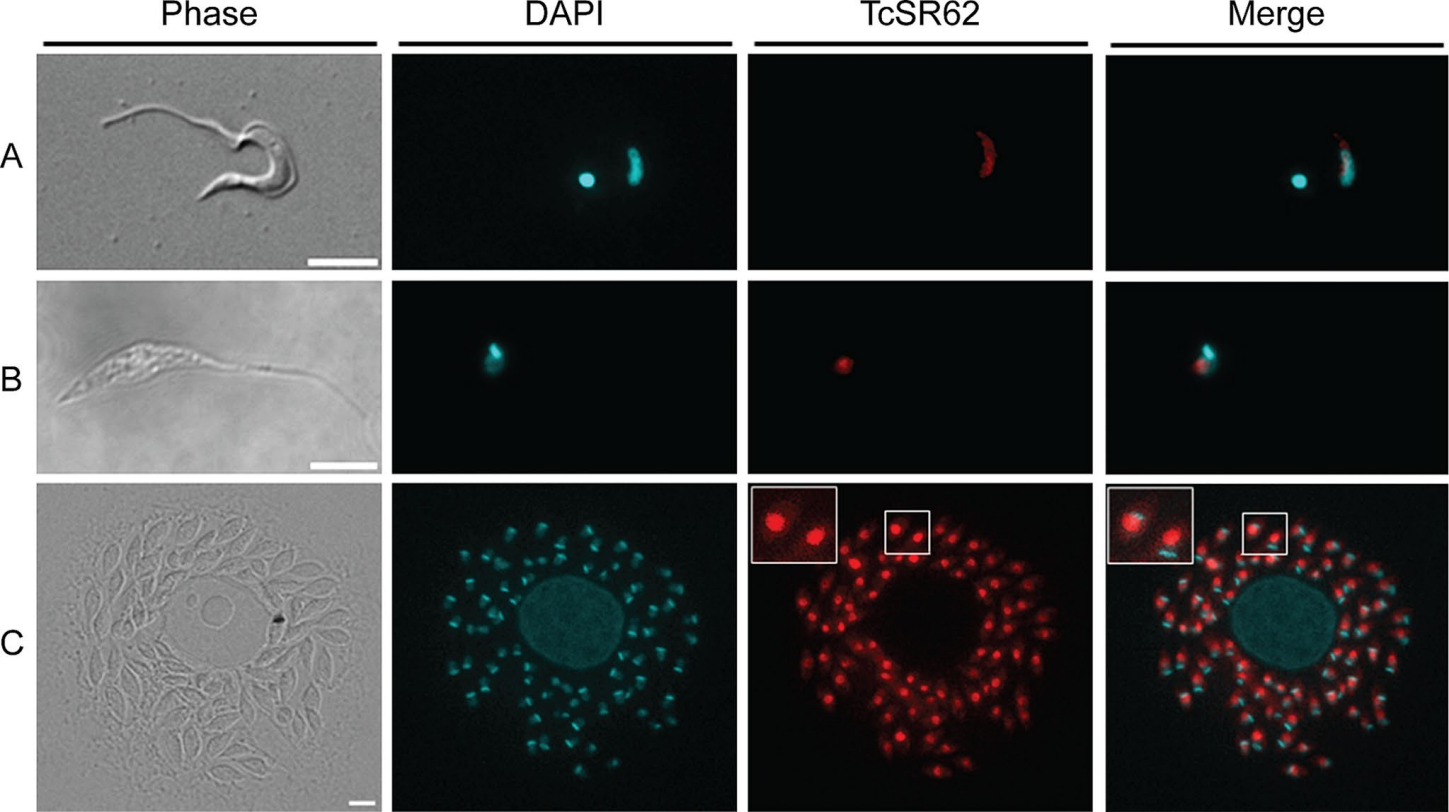
Subcellular localization of TcSR62. Indirect immunofluorescence photographs of trypomastigotes **(A)**, epimastigotes **(B)**, and amastigote stages **(C)** of the Dm28c parasites. The slides were stained with DAPI to visualize nuclei and kinetoplasts. The microphotographs were analyzed using a Nikon Eclipse 80i microscope with the appropriate filters. Scale bar: 5 μm.

To evaluate the subcellular localization of TcSR62, indirect immunofluorescence assays (IIF) were conducted on trypomastigotes, epimastigotes and amastigotes from distinct strains. The results revealed that the TcSR62 protein was primarily localized in the nucleus of both trypomastigotes and epimastigotes of the Dm28c strain (Figures 2A,B). Similar results were obtained for trypomastigotes of the CL-Brener, RA and Sylvio strains (Supplementary Figure S1A) and in CL-Brener and Y epimastigotes (Supplementary Figure S1B). Experiments on Dm28c amastigotes revealed that the TcSR62 protein was predominantly localized in the nucleus of parasites, with a weak fluorescence signal detected in the cytoplasm (Figure 2C, and inset). Comparable findings were observed in amastigotes of CL-Brener, RA, Y, and Tul β-gal strains (Supplementary Figure S1C). The consistent nuclear localization of TcSR62 across various strains and life stages strongly supports its involvement in nuclear processes within *T. cruzi*.

### 3.3 TcSR62 molecular modeling and docking

**Figure 3:**
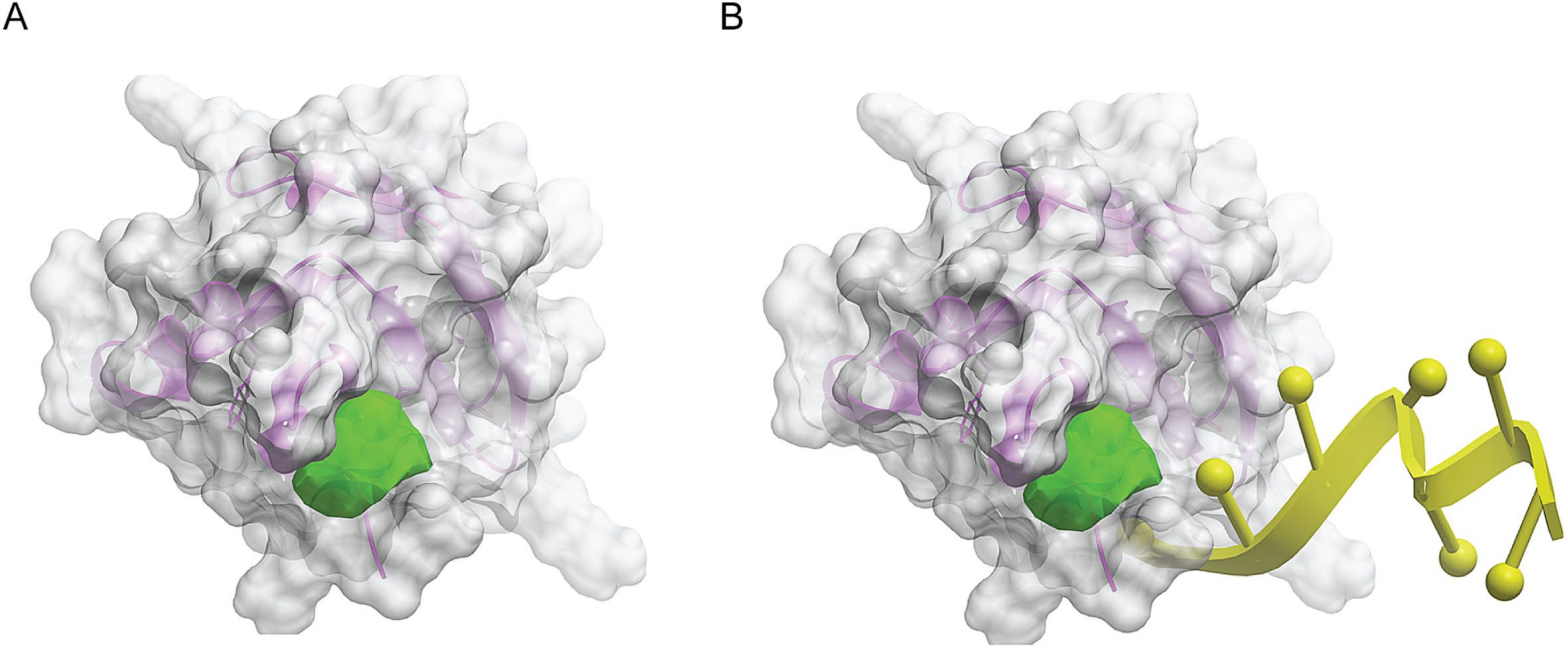
Molecular modeling of the first RRM domain of TcSR62 protein. Homology model of the RRM1 domain of TcSR62 (magenta ribbon depiction of the protein backbone and transparent gray mesh depiction of the protein surface) with chosen pocket (green geometric object) for library docking **(A)**. Model showing the location of the hnRNA (yellow) in the parent homologous structure, which would be the predicted location of parasitic RNA binding **(B)**.

**Figure 4:**
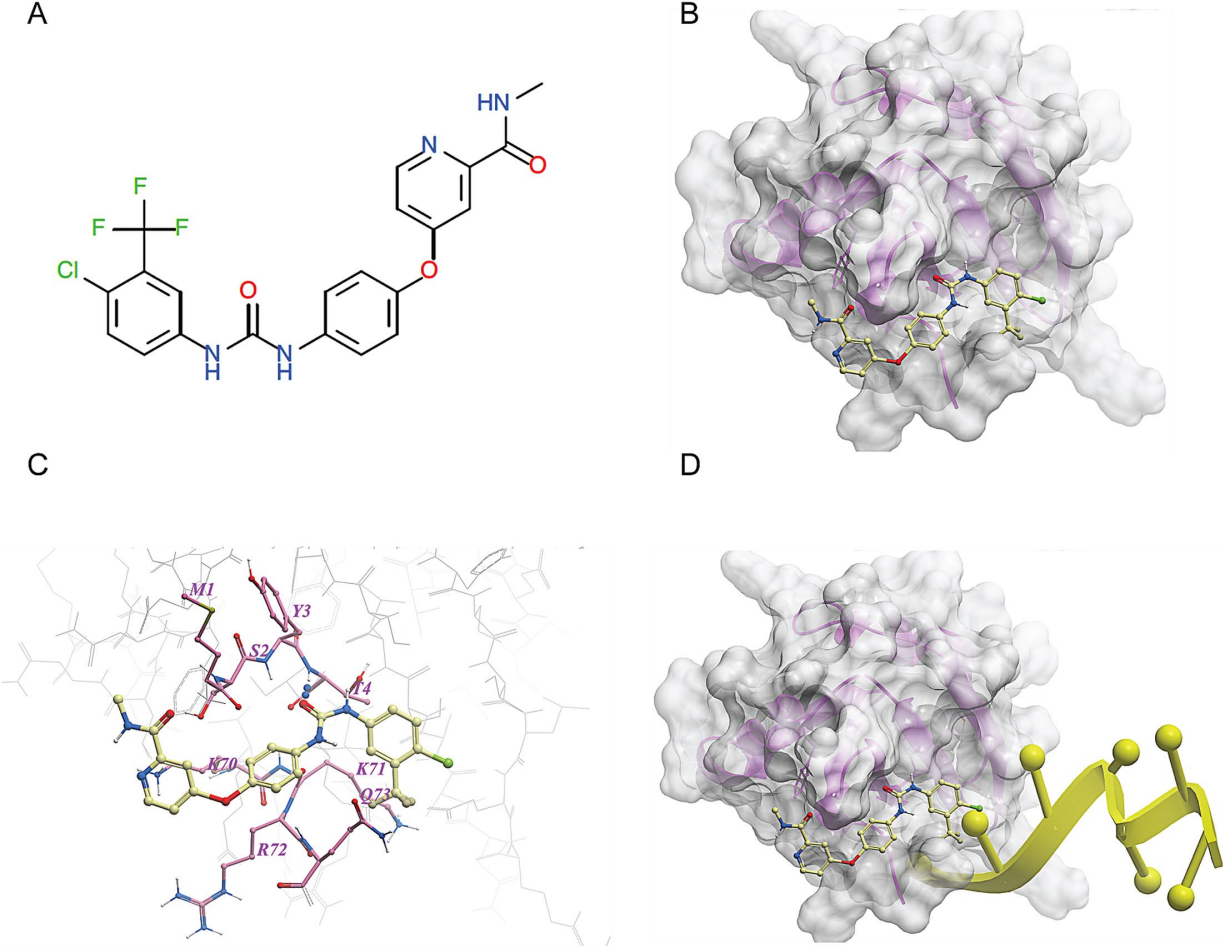
Molecular docking of sorafenib tosylate in the RRM1 domain of TcSR62. **(A)** Chemical structure of sorafenib tosylate (ST). **(B)** Model of the molecular surface of the RRM1 domain of TcSR62 (transparent gray mesh) with docked ST (yellow ball-and-stick depiction of atoms and bonds). **(C)** Map of contact points between TcSR62 atoms (gray wire depiction for unlabeled, non-contacting amino acids and pink stick depiction for labeled, contacting amino acids) and ST (yellow stick depiction). **(D)** Model of the molecular surface of the RRM1 domain of TcSR62 (transparent gray mesh) with docked ST (yellow stick depiction) showing impingement at right end with predicted location of bound parasitic RNA (yellow ribbon depiction).

We made a 3D structural model of the *T. cruzi* TcSR62 RRM domains based on a published crystallographic structure of an RRM domain of hnRNPA2/B1 protein bound to RNA (PDB ID 5en1). The target drug binding pocket (volume = 115.5 angstroms) was found in an area of the domain that binds RNA in the homologous RRM1 structure (Figures 3A,B) (Wu et al., 2018). Accordingly, we computational screened a library of U.S. FDA approved drugs against this potential drug-binding pocket in this structural model, with the hypothesis that compounds with excellent calculated biophysical compatibility with this pocket could bind to the pocket and competitively inhibit binding, and therefore possibly processing of RNA in parasites. Table 1 summarizes the list of drugs with high predicted biophysical complementarity to the RRM1 pocket. Sorafenib tosylate (ST) was selected as a candidate drug for further testing (Figure 4A). This compound binds to the selected pocket with a calculated energy of −19.20 (Table 1; Figures 4B,C) in the area where RRM1 binds the RNA (Figure 4D).

### 3.4 Sorafenib tosylate showed the strongest trypanocidal effect

**Figure 5:**
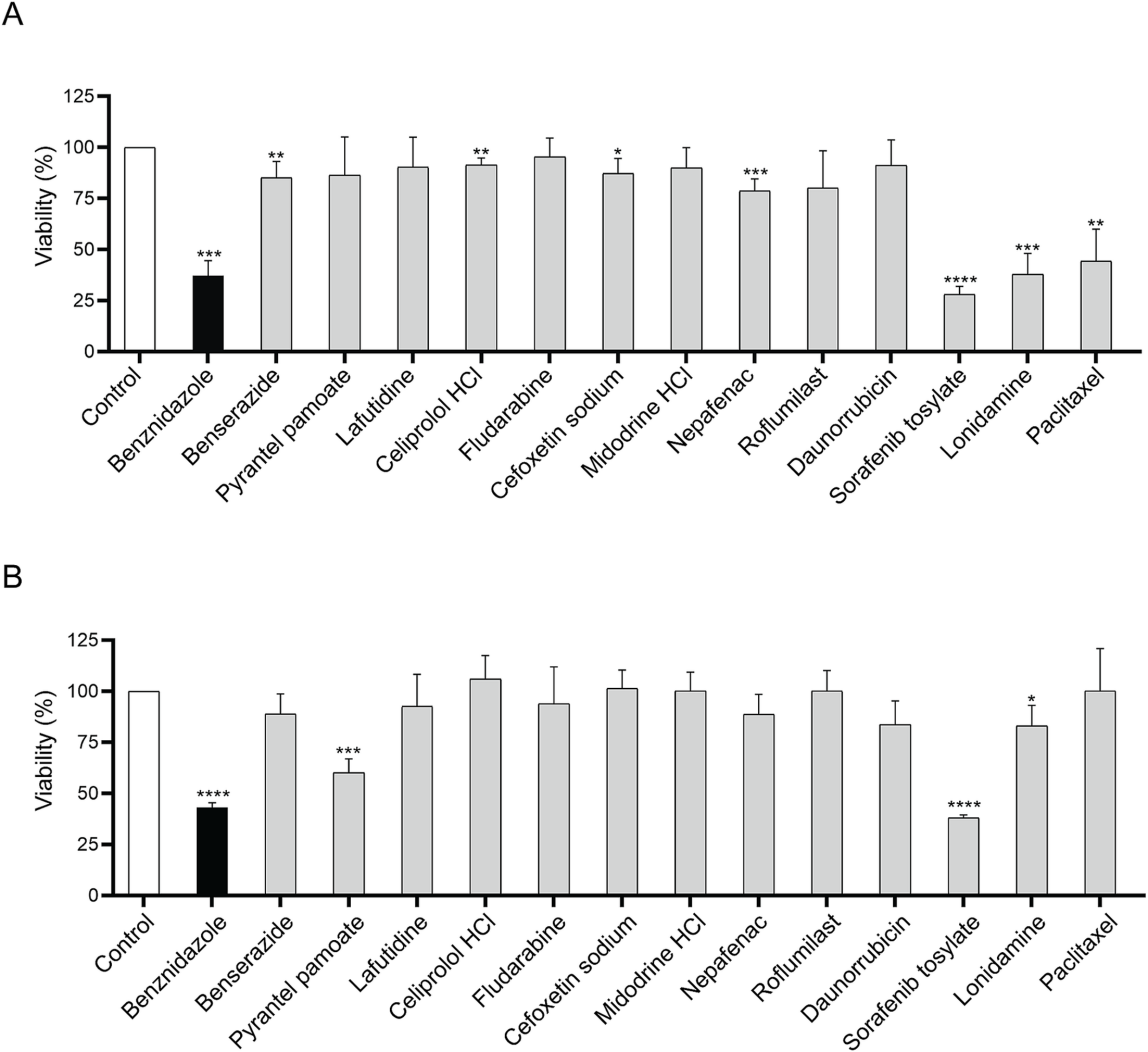
Parasite viability after 10 μM incubation with candidate drugs. Viability assays in CL-Brener trypomastigotes **(A)** and in Y epimastigotes **(B)**. Viability was performed by adding resazurin and measuring fluorescence at 595 nm. Each drug was compared to its corresponding vehicle (H_2_O or 0.5% DMSO). Unpaired Student’s *t*-test. Error bars indicate the standard deviation of at least three biological replicates. *p < 0.05; **p < 0.01; ***p < 0.001; ****p < 0.0001.

Candidate drugs were assayed for the viability of CL-Brener trypomastigotes and Y epimastigotes. In the trypomastigote stage, ST demonstrated strong trypanocidal activity, resulting in a cell viability of 28.2% ± 3.87% after 24 h at 10 μM final concentration (Figure 5A). Similar results were observed after incubation with lonidamine which caused a decrease in parasite viability to 37.84% ± 10.38%. On the other hand, after 10 μM Pyrantel pamoate or ST, viability reached to 60.1% ± 6.9 and 38.2% ± 1.4%, respectively after 48 h of incubation in epimastigotes (Figure 5B). These data indicate that ST presented the highest trypanocidal activity in both trypomastigotes and epimastigotes.

### 3.5 Sorafenib tosylate showed a potent half maximal inhibitory concentration

**Figure 6:**
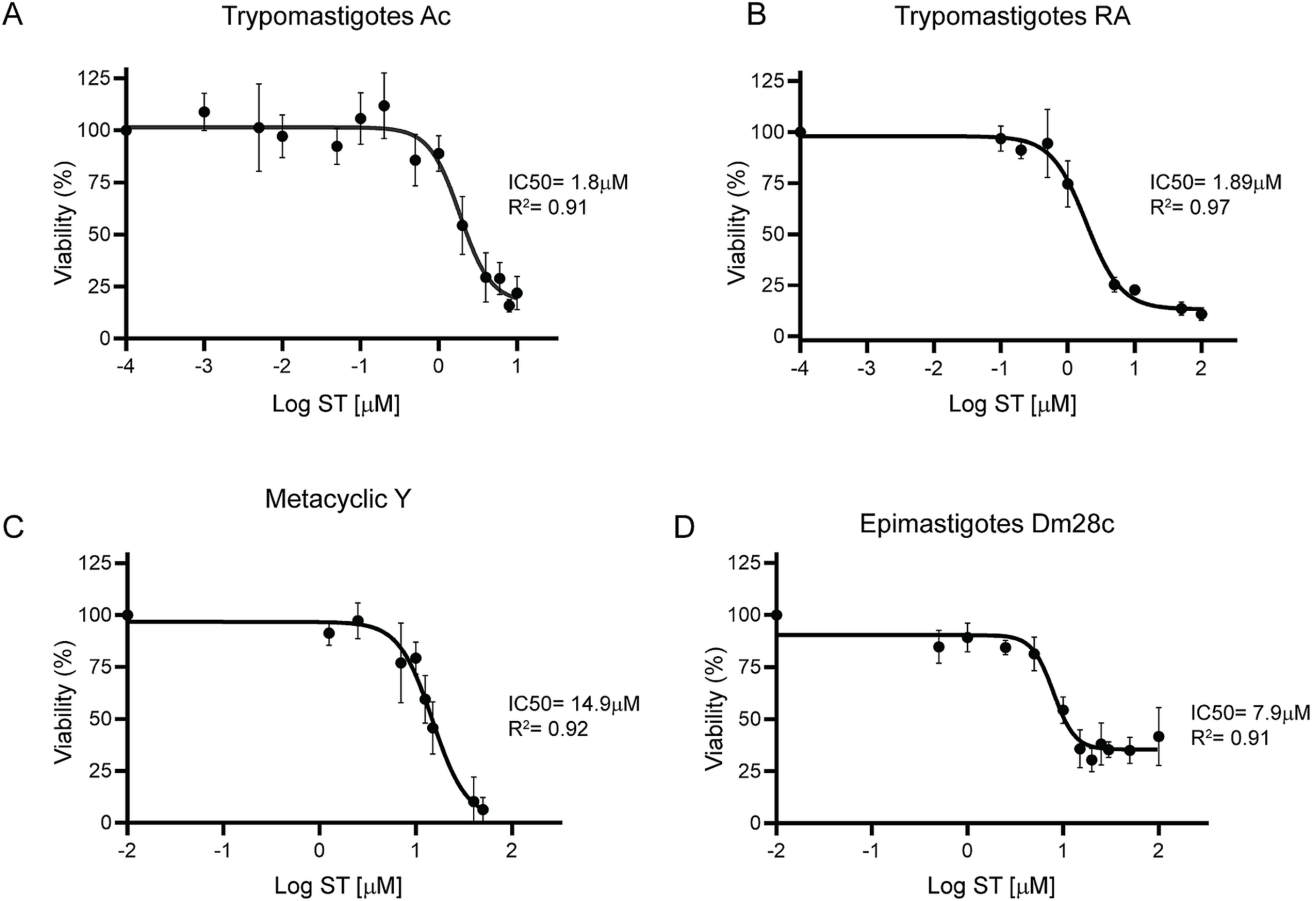
Dose–response curves of sorafenib tosylate in different *T. cruzi* stages. Viability was determined for the Ac **(A)** and RA **(B)** cell-derived trypomastigotes, metacyclic-stage Y parasites obtained from aged trypomastigotes **(C)** and Dm28c epimastigotes **(D)**. The cell-derived trypomastigote and epimastigote viability percentages were determined by resazurin assay and fluorescence measurement at 595 nm. The percentage of metacyclic parasites was determined by counting in a Neubauer chamber in the presence of different ST concentrations. The percentages were normalized to 0.5% DMSO. The results were obtained from at least three independent biological replicates. The IC50 was calculated with GraphPad Prism 9 and adjusted to a dose–response curve using the variable slope model (four parameters).

**Figure 7:**
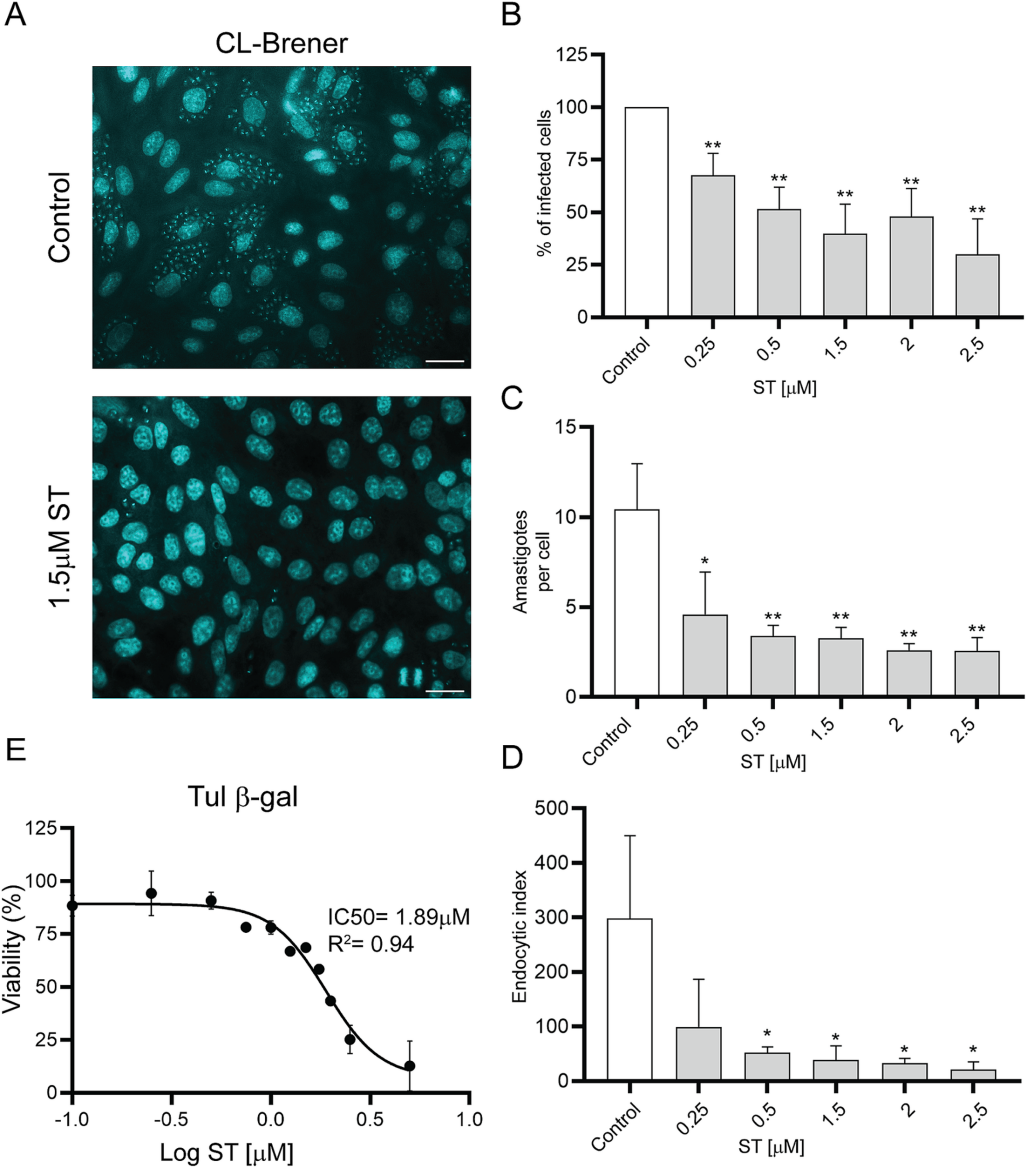
Effects of sorafenib tosylate on amastigote-stage parasites. **(A)** DAPI staining of Vero cells infected with CL-Brener trypomastigotes in the presence of ST. The coverslips were stained with DAPI for visualization under a fluorescence microscope. Scale bar: 50 μm. Graphs show percentage of infected cells **(B)**, number of amastigotes per cell **(C)**, and endocytic index **(D)** after treatment with ST. The percentage of infected cells was normalized to the vehicle control (0.5% DMSO). Results are shown from three independent biological replicates, with their respective standard deviation, counting at least 900 cells per treatment. *p < 0.05; **p < 0.01. **(E)** Viability for the Tul β-gal strain using the resazurin assay. The IC50 was calculated using GraphPad Prism 9 and adjusted to a dose–response curve using the variable slope model (four parameters).

To determine the 50% inhibitory concentration (IC50) of ST, cell-derived trypomastigotes, metacyclic trypomastigotes and epimastigotes were incubated with different concentrations of ST for 24 h. Sorafenib tosylate showed an IC50 value of 1.8 μM and 1.89 μM in trypomastigotes of Ac and RA strain parasites, respectively (Figures 6A,B). On the other hand, in Y strain metacyclic trypomastigotes, ST showed an IC50 of 14.9 μM (Figure 6C), whereas in epimastigotes of Dm28c an IC50 of 7.9 μM ST was observed (Figure 6D). Additionally, to determine the trypanocidal effect of ST on amastigotes, Vero cells infected with the CL-Brener strain were used, and the percentage of infected cells, number of amastigotes per cell, and endocytic index were determined at different concentrations of ST. The results showed that ST produced a significant decrease in the percentage of infected cells from 0.25 μM (67.78% ± 10.28%) to 2.5 μM (30.03% ± 16.9%) after 72 h of incubation (see Figures 7A,B). Sorafenib tosylate also produced a decrease in the number of amastigotes per cell, reaching from 4.6 ± 2.3 (0.25 μM) to 2.5 ± 0.73 (2.5 μM) compared to control (10.43 ± 2.5) (Figure 7C). Because infected cells have a variable number of amastigotes, the endocytic index was calculated. Sorafenib tosylate caused a significant reduction in the endocytic index from 0.5 μM ST yielding a value of 52.27 ± 10.1 compared to control (298.4 ± 151.7) (Figure 7D). To corroborate these results, Vero cells infected with Tulahuen trypomastigotes expressing β-galactosidase (Tul β-gal) were incubated with different concentrations of ST. Results showed an IC50 = 1.89 μM indicating that ST produced a profound decrease in parasite viability (Figure 7E). These findings confirm that ST exhibited potent trypanocidal activity across the entire life cycle of *T. cruzi*, significantly reducing parasite viability, infection rates, and the number of intracellular amastigote cells at low micromolar concentrations.

### 3.6 Sorafenib tosylate showed a promising selectivity index in H9C2 cells

**Figure 8:**
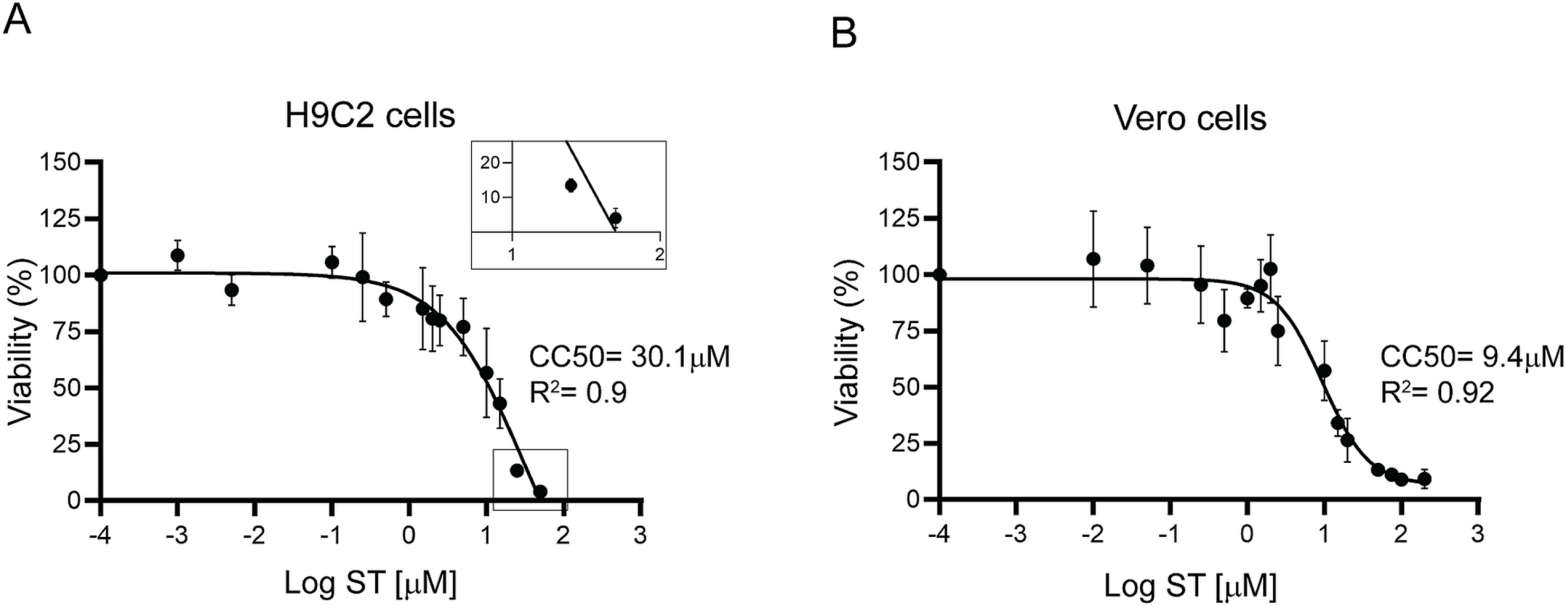
Cytotoxicity of sorafenib tosylate. Cytotoxicity was determined by resazurin viability assay in H9C2 cardiomyocytes **(A)** and Vero cells **(B)** after 72 h of incubation with different concentrations of ST. Results from at least three independent biological replicates are shown with their respective standard deviations. The CC50 was calculated using GraphPad Prism 9, adjusting to a dose–response curve using the variable slope model (four parameters). The inset in **(A)** shows ST toxicity on an enlarged scale, magnifying the lower end of the curve.

To evaluate ST cytotoxicity, the cardiomyocyte cell line (H9C2) and Vero cells were used. The cells were incubated for 72 h with different concentrations of ST and viability was measured by determining the absorbance of resazurin at 570 nm. The H9C2 cell line exhibited a half-maximal cytotoxic concentration (CC50) of 30.1 μM (Figure 8A), whereas Vero cells showed a CC50 of 9.4 μM (Figure 8B). The selectivity index resulted in 16.7 for H9C2 cells and 5.22 for Vero cells. These results indicate that ST was less toxic in H9C2 cells than in Vero cells, which resulted in a higher selectivity index in cardiac cells compared to Vero cells.

### 3.7 TcSR62 overexpression led to sorafenib tosylate resistant parasites

**Figure 9:**
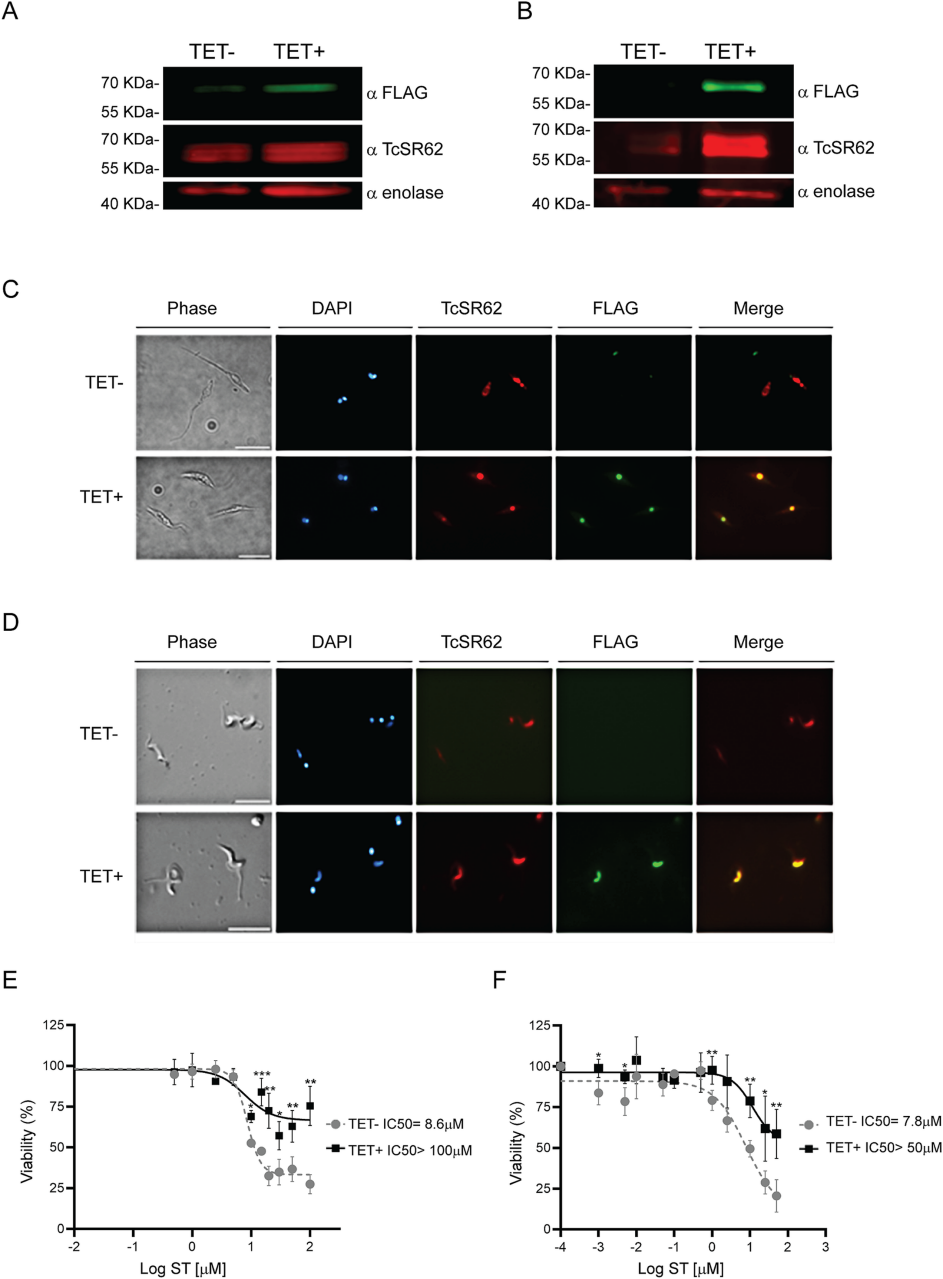
Overexpression of 3xFLAG-TcSR62 and sorafenib tosylate IC50 determination. Western blot in epimastigote parasites **(A)** and trypomastigote parasites **(B)** after 24 or 3 h of TET addition, respectively. TcSR62 detection was performed using a serum that specifically recognizes TcSR62 protein. For overexpression, an anti-FLAG monoclonal antibody was used and anti-enolase serum was used as loading control. Protein detection was performed using the Odyssey DLx Imaging System (LI-COR Biosciences). IIF in epimastigote **(C)** and trypomastigote **(D)** parasites after TET induction. The slides were stained with DAPI to visualize the nuclei and kinetoplasts. The microphotographs were analyzed using a Nikon Eclipse 80i microscope with the appropriate filters. Scale bar: 10 μm. The IC50 in epimastigote **(E)** or trypomastigote **(F)** stage parasites overexpressing 3xFLAG-TcSR62 compared to uninduced cultures is shown. The IC50 was calculated using GraphPad Prism 9, adjusting to a dose–response curve using the variable slope model (four parameters). Unpaired Student’s *t*-test was used to compare TET- and TET+ cultures. Error bars indicate the standard deviation of at least three biological replicates. *p < 0.05; **p < 0.01; ***p < 0.001.

To evaluate whether the effect of ST was related to TcSR62 protein function, parasites conditionally expressing the 3xFLAG-TcSR62 protein were obtained. After TET induction, 3xFLAG-TcSR62 expression was studied. Western blot assay showed higher expression levels in both TET+ epimastigotes and trypomastigotes compared to uninduced cultures (Figures 9A,B, respectively). IIF assays showed complete nuclear colocalization of TcSR62 protein and its induced expression version in epimastigotes (Figure 9C) and in trypomastigotes (Figure 9D). To evaluate the IC50 of ST in TET− and TET+ cultures, parasite viability was studied after incubation with different concentrations of the compound. Results show in non-induced epimastigotes an ST IC50 = 8.6 μM while after TET addition, IC50 was >100 μM (Figure 9E). Regarding the trypomastigote stage, in TET- cultures the IC50 value was 7.8 μM and an IC50 > 50 μM was obtained in parasites overexpressing TcSR62 protein (Figure 9F). In conclusion, the IC50 of ST increased more than 11-fold in epimastigote and 6-fold in trypomastigote parasites overexpressing the 3xFLAG-TcSR62 protein compared to the non-induced culture. These results suggest that the trypanocidal effect of ST could be effectively associated with TcSR62 protein function, as its overexpression produced resistant parasites, as evidenced by a strong increase in the IC50 value in TET+ parasite cultures.

## 4 Discussion

In contrast to other diseases caused by trypanosomatids, for which most current chemotherapies are repurposed medications, drug repurposing strategies for CD have shown limited success and many of them failed during *in vivo* studies (Porta et al., 2023; Ramírez-Macías et al., 2024). However, repurposing existing drugs and adjusting dosing schedules represent the most rapidly deployed approaches for enhancing CD management. These methods can significantly reduce the costs and duration associated with the development of new medications, as they may already have established pharmacokinetic and safety profiles (García-Huertas and Cardona-Castro, 2021). In recent years, considerable efforts were directed toward discovering new therapeutic strategies, especially for the chronic stage (Ramírez-Macías et al., 2024). The development of new drugs with trypanocidal activity for treating CD is crucial, as currently, only one compound, a benzoxaborole derivative (DNDI-6148, CPSF3 inhibitor), has reached phase I in clinical trials (Padilla et al., 2022; Tarleton, 2023).

In this work, we identified ST as a compound with a potential interaction with the first RRM of TcSR62 (Figure 4; Table 1), an RBP of *T. cruzi* known to be expressed in the nucleus of the epimastigote stage form of the CL-Brener strain (Názer et al., 2011). First, we characterized the expression profile of TcSR62 protein in different strains and stages of *T. cruzi* parasite. Experiments using western blot assays showed that TcSR62 is expressed in trypomastigotes, epimastigotes and in the amastigote stage of CL-Brener, Sylvio, RA, Dm28c and Ac strains of *T. cruzi* (Figure 1), belonging to different parasite DTUs. IIF assays showed a major nuclear TcSR62 localization in all stages of Dm28c and other strains (Figure 2; Supplementary Figure S1) with a minor localization in the cytoplasm of amastigotes, indicating that TcSR62 is widely expressed in all stages of *T. cruzi* and suggesting additional roles of TcSR62 protein in the amastigote stage (Figure 2C; Supplementary Figure S1C). Considering this result, it’s tempting to speculate that TcSR62 function in amastigotes could also be related to mRNA stability.

Then, ST trypanocidal activity was analyzed in cell-derived trypomastigotes, in metacyclic parasites, in epimastigotes and in the amastigote stage. The lowest ST IC50 values were observed in the mammal specific stages (trypomastigotes and amastigotes, Figures 6A,B, 7E) which is particularly promising since ST can be considered as a potential therapeutic option.

Sorafenib tosylate has been approved for clinical use in the treatment of advanced-stages of renal cell carcinoma (Yang et al., 2012) and hepatocellular carcinoma (Llovet et al., 2008). It inhibits cell proliferation and induces cell death in various cancer cell lines through different mechanisms, including apoptosis and ferroptosis. The effectiveness of ST varies across different cell lines, with some being more sensitive than others (Liu et al., 2006; Lachaier et al., 2014). It is important to note that while ST shows efficacy against these cell lines *in vitro*, its effectiveness in clinical settings may differ owing to various factors, including drug resistance mechanisms (Zhu et al., 2017).

Since biophysical drug binding affinity is commonly 10-fold higher than observed cellular IC50 (Knight and Shokat, 2005), we estimate the affinity of ST to the TcSR62 domain to be approximately 200 nM. Because ST is a multi-target kinase, this affinity is in the same range as ST’s inhibition of a variety of human kinases, including several important MAPKs and CDKs (from LINCS database; Keenan et al., 2018). Sorafenib tosylate was initially designed as an FGFR1 suppressor but found to bind promiscuously to numerous serine/threonine and tyrosine kinases, including RAF1, BRAF, VEGFR, PDGFR, KIT, FLT3, and RET (Gong et al., 2017). Given ST’s propensity to bind off-targets it is not surprising that ST could cross react with a distant pocket like that seen in TcSR62. In this report, we show that ST fits well biophysically to a pocket on the RRM1, suggesting a possible anti-trypanosomal mechanism of action by interacting with the RNA-binding domain and disrupting the binding of endogenous targets, thereby inhibiting *T. cruzi* survival. Since ST is already in clinical use and exhibits predictable pharmacology and toxicity in humans, this makes it an ideal repurposed drug.

Sorafenib tosylate has been shown to be a trypanocidal agent in other parasite species. In axenic *T. evansi* (Steel, 1885; Chauvrat, 1896) parasites, ST showed trypanocidal activity at 5 μM after 24 h of incubation in a dose-dependent and time-dependent increase of ROS generation (Kumar et al., 2020). On the other hand, in the bloodstream form of *T. brucei*, ST was identified as a TbGSK3β protein inhibitor involved in the transferrin endocytosis (Guyett et al., 2016). Consequently, these data show that ST is a promising candidate for the development of anti-trypanosomal treatments.

To corroborate that ST trypanocidal activity was related to the TcSR62 protein, Dm28c parasites overexpressing a tagged version of this protein were incubated with different concentrations of the compound. The ST IC50 obtained from TET+ cultures significantly increased, with trypomastigotes and epimastigotes tolerating ST concentrations exceeding 50 μM and 100 μM, respectively (Figures 9E,F). These data suggest that the trypanocidal effect of ST may be linked to the function of TcSR62 protein; either directly, by inhibiting the RNA binding function of RRM1 or indirectly, by affecting the protein interaction networks that this RBP may establish. The IC50 observed in TET− Dm28c pLew cultures for epimastigotes (Figure 9E) was only slightly higher than that of the parental cell line (8.6 μM compared to 7.9 μM in Figure 6D). However, in trypomastigotes, the TET− IC50 value for pLew cells reached 7.8 μM, which was approximately 4 times greater than that of the wild type lines. This discrepancy may be attributed to the leaky basal expression inherent in this inducible system (Taylor and Kelly, 2006; Alonso et al., 2014), which was not detectable by western blot analysis.

In Vero cells, the selectivity index was 5.22, whereas in H9C2 cardiac cells the selectivity index was higher than 16, which is considered to be specific for antiparasitic compounds (Peña et al., 2015). On the other hand, ST has demonstrated a generally well-tolerated safety profile across multiple studies and cancer types. The most common drug-related toxicities are skin reactions, rash, diarrhea, nausea, hypertension and fatigue (Takimoto and Awada, 2008; Li et al., 2014), which are typically mild to moderate in severity and can be managed with appropriate strategies (Keating, 2017).

In conclusion, we observed that sorafenib tosylate has a potent trypanocidal effect and that the TcSR62 protein is an essential factor of ST trypanocidal action. As TcSR62 has no orthologs in mammals and possesses domains that may be inhibited by drug-like compounds, this study highlights its potential as a novel target for therapeutic strategies against CD.

## Data Availability

The original contributions presented in the study are included in the article/Supplementary material, further inquiries can be directed to the corresponding author.

## Supplementary material

Figure S1 Subcellular localization of TcSR62 in different *T. cruzi* strains and stages. Indirect immunofluorescence images of *T. cruzi* trypomastigotes from the CL-Brener, RA, and Sylvio strains **(A)**; epimastigotes from the CL-Brener and Y strains **(B)**; and amastigotes from the CL-Brener, RA, Y, and Tul β-gal strains **(C)**. The slides were stained with DAPI to visualize nuclei and kinetoplasts. Images were captured and analyzed using a Nikon Eclipse 80i microscope equipped with the appropriate fluorescence filters. Scale bar: 5 μm.
