# Supplementary figures and images for: TcSR62, an RNA-binding protein, as a new potential target for anti-trypanocidal agents
Source: Front Microbiol. 2025 Mar 12;16:1539778. doi: 10.3389/fmicb.2025.1539778 (PMC11936972; doi:10.3389/fmicb.2025.1539778)

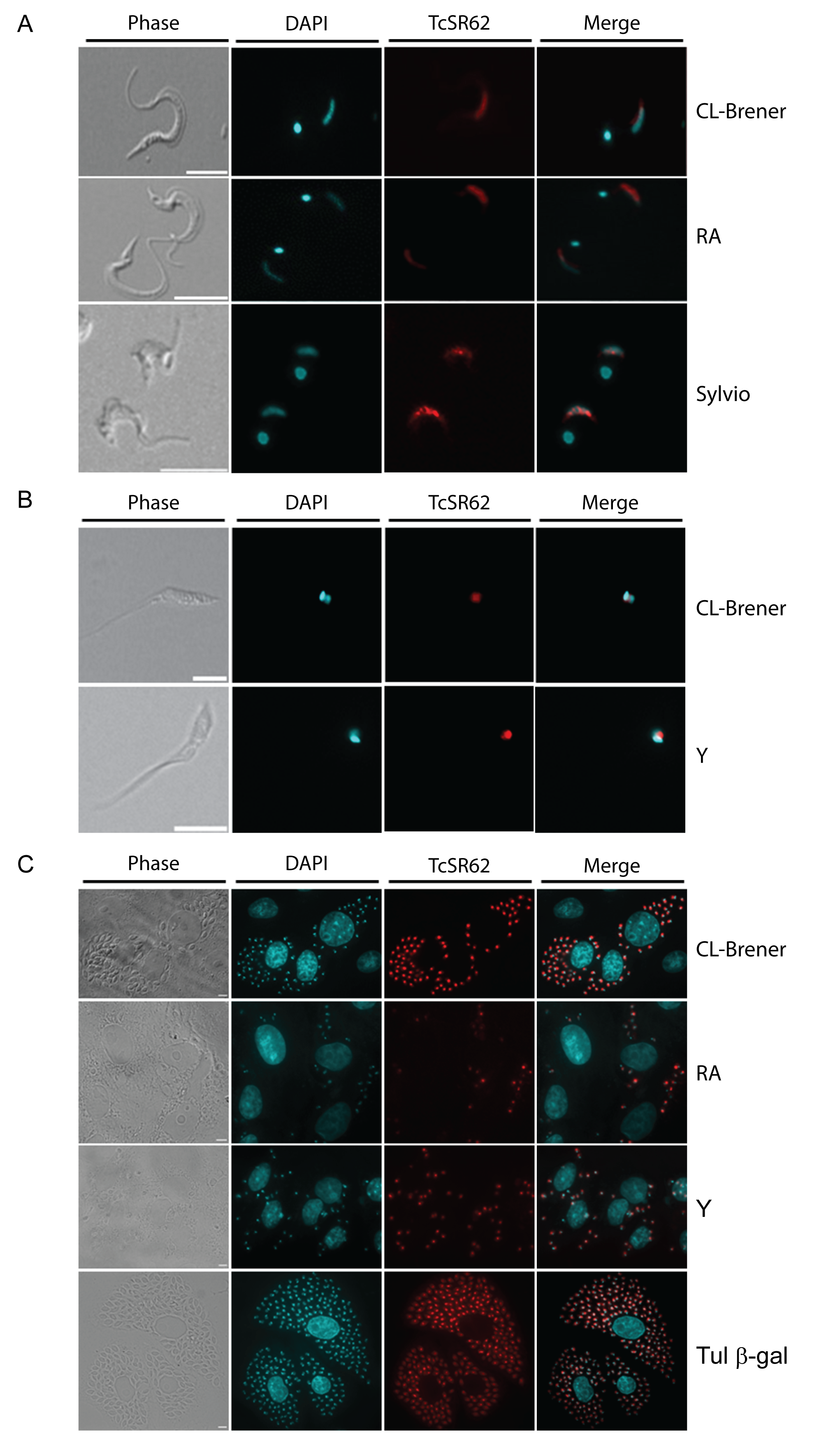

Supplement: Figure S1 — Subcellular localization of TcSR62 in different T. cruzi strains and stages. Indirect immunofluorescence images of T. cruzi trypomastigotes from the CL-Brener, RA, and Sylvio strains (A); epimastigotes from the CL-Brener and Y strains (B); and amastigotes from the CL-Brener, RA, Y, and Tul β-gal strains (C). The slides were stained with DAPI to visualize nuclei and kinetoplasts. Images were captured and analyzed using a Nikon Eclipse 80i microscope equipped with the appropriate fluorescence filters. Scale bar: 5 μm. [file Image_1.tif]
